# Supplementary material for: Drip irrigation and sulphur fertilization influenced fodder yield, quality and water use efficiency of groundnut in arid region
Source: PLoS One. 2023 Aug 4;18(8):e0288090. doi: 10.1371/journal.pone.0288090 (PMC10403097; doi:10.1371/journal.pone.0288090)
Supplement: Attachment — Submitted filename: Response to Reviewer comments.docx [file pone.0288090.s001.docx]

**Journal Name:** PLOS ONE

**Manuscript title**: Drip irrigation and sulphur fertilization influenced fodder yield, quality and water use efficiency of groundnut in arid region

**Ms. Ref. No**.: PONE-D-23-01558

**Dear Editor**,

We are very thankful to the reviewers’ for useful comments and suggestions on the structure of our manuscript. We are very also very thankful for thoroughly reviewing our manuscript. These comments and suggestions were helpful in improving our manuscript and we have addressed them in the revised version in track change mode.

Here we are also providingdetailed responses to the reviewers’ comments.

| **S. No.** | **Comments** | **Response** |
| --- | --- | --- |
|  | **Reviewer 1** | |
| 1. | Your paper "Drip irrigation and sulphur fertilization influenced fodder yield, quality and water use efficiency of groundnut in arid region" addresses the fodder quality of groundnut. Problem of fodder deficiency and quality is severe in arid regions. Paper is written well. Introduction describes a clear background and significance of the problem to be solved. Methodology is described thoroughly. Results and discussion section written well.  However, it needs to be revised to check spell mistakes and for language improvement. In conclusion, only a few lines are enough describing key findings of the study. | We want to thank reviewers for appreciating our reports.  Agreed and complied;  Manuscript is revised for language and spellings, extra lines were deleted from conclusion. |
|  | **Reviewer 2** | |
| 2. | Line 14: “However, no researchershavegiven emphasis” insert space between words. | Agreed and complied |
| 3. | Line 22: Define PE in Abstract. | Agreed and complied |
| 4. | Line 58: “20-22 irrigation” Author means no. of irrigations? Too high? Cite it if yes, please! | corrected the line and reference is provided for rest sentence |
| 5. | Line 62: “regions of India.Drip irrigation” insert space after full stop | Agreed and complied |
| 6. | Line 115: Define FIR? | It is a seed treatment sequence of fungicide, insecticide, Rhizobium  Defined in text also |
| 7. | Line 185: Typo error in unit of WUE. | Agreed and complied, unit is changed accordingly |
| 8. | Line 202: Table 1, Add letters to show significant differences between treatment means. | Agreed and complied |
| 9. | Line 233: Table 2, Add letters to show significant differences between treatment means. | Agreed and complied |
| 10. | Line 258: Table 3, Add letters to show significant differences between treatment means. | Agreed and complied |
| 11. | Line 273: Table 4, Add letters to show significant differences between treatment means. | Agreed and complied |
| 12. | Line 275: Table 5, Add letters to show significant differences between treatment means. | Agreed and complied |
| 13. | Line 305: Table 6, Add letters to show significant differences between treatment means. | Agreed and complied |
| 14. | Line 332: Table 7, Add letters to show significant differences between treatment means. | Agreed and complied |
| 15. | Line 336: “Effect drip irrigation” Effect of drip irrigation | Agreed and complied |
| 16. | Line 379: “Effect drip irrigation” Effect of drip irrigation | Agreed and complied |
| 17. | Line 426: “0-60 kg ha-1” 0-60 kg ha-1 | Agreed and complied |
|  | **Reviewer 3** | |
| 1. | The manuscript needs to be further proof read to improve the English further. Few grammatical errors or omissions such as in line 1, 38, 49, 92-93, 194, 222-223, 352, 369 etc can be avoided. | Agreed and complied;  Manuscript is revised for language and spellings for improving the English. |
| 2. | Please include the reference to the point raised in line 77-79, ‘In most of the 78 groundnut growing tracts, the level of available sulphur reaches below the critical limit and groundnut crop is bound to suffer on account of sulphur deficiency. | Agreed and complied;  Reference is added |
| 3. | In line 95-97, the data presented was minimum to maximum rather than maximum to minimum e.g ‘The maximum and minimum temperature ranged between 26.6℃ to 43.2℃ and 8.5℃ to 31.1℃ | Agreed and complied; |
| 4. | It will be nice if the authors present the initial soil test of the location used. Authors can provide them if available | Agreed and complied;  Initial soil data was provided in the materials and methods section |
| 5. | In your statistical analysis, please confirm that the data followed normal distribution and equal variances, which justifies the use of ANOVA. | The data is having normal distribution and equal variances, same is mentioned in the text also |
| 6. | In your results and tables, I will prefer you include the exact P values for the main effects and the interactions. This will make comparison easier. | Agreed and complied  CD was already provided in the tables at 5% level of significance, now letters were also added to show the significant differences between treatment means |
| 7. | When reporting the specifics in the first few tables, you did not mention which planting year you are referring to. One can only generally assume that both years followed the same trend, but this was also not mentioned by the authors. | Table 1, 6 and 7 data of both the years presented and in table 2 & 4, data of 2020; in table 3 & 5, data of 2021 was provided and same was mentioned in table title/heading. |

**Priyanka Gautam**

Corresponding author
